# Successful retrieval of a fractured migrated pancreatic stent using an endoscopic tapered sheath for severe pancreatic duct stenosis

**DOI:** 10.1055/a-2086-1946

**Published:** 2023-05-26

**Authors:** Akira Higashimori, Hirotsugu Maruyama, Natsumi Maeda, Yuki Ishikawa-Kakiya, Masafumi Yamamura, Kojiro Tanoue, Yasuhiro Fujiwara

**Affiliations:** Department of Gastroenterology, Osaka Metropolitan University Graduate School of Medicine, Osaka, Japan

Endoscopic pancreatic stenting is a well-accepted treatment for symptomatic pancreatic duct stenosis 1 2 ; however, during stent removal, stent fracture can occur owing to severe pancreatic duct stenosis 3 4 . In such cases, removal of the migrated fractured stents is difficult using conventional devices, which are unable to pass the site of the severe stenosis over the guidewire. Therefore, we used an endoscopic tapered sheath (EndoSheather; Piolax Inc., Kanagawa, Japan), a device that simultaneously enables both stenosis breakthrough and retrieval of the migrated stent (up to a diameter of 1.9 mm) through the indwelling outer sheath ( Fig. 1 ).

**Fig. 1:**
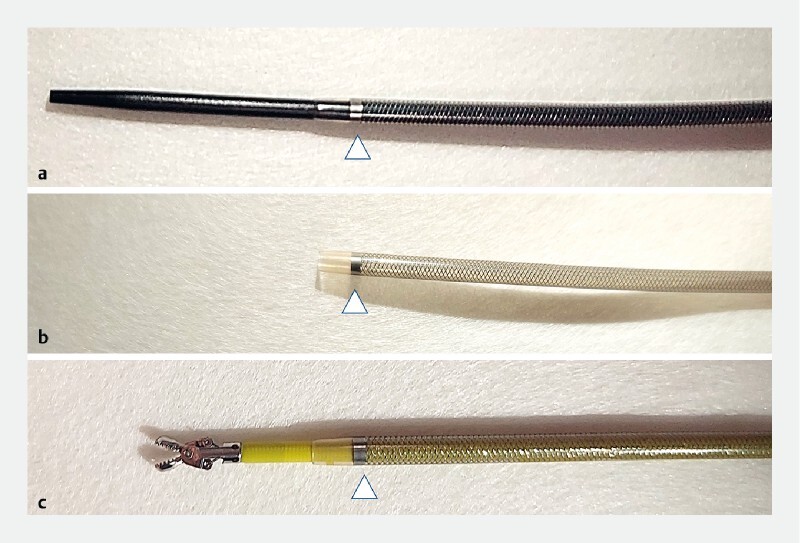
Photographs of the endoscopic tapered sheath (EndoSheather) showing: **a** it is composed of a tapered inner catheter (2.06-mm diameter; 6.2 Fr) and an outer sheath (2.44-mm diameter; 7.2 Fr) with a radiopaque marker (white arrowhead), with these components together serving as a dilator for any stenosis; **b** with the inner catheter removed, the outer sheath can act as a conduit for the insertion of various devices up to a diameter of 1.9 mm (5.7 Fr); **c** pediatric biopsy forceps with a diameter of 1.8 mm (Radial Jaw 4 pediatric biopsy forceps) inserted through the sheath.

A 55-year-old man who had undergone placement of a 5-Fr stent 3 months previously for a severe stenosis of the pancreatic duct from the head to the body that had been caused by chronic pancreatitis ( Fig. 2 ) was admitted for a stent change. The stent fractured during removal ( Fig. 3 ) and it could not be retrieved because the stenosis did not allow the passage of any conventional retrieval devices. We therefore attempted to retrieve the fractured stent using an endoscopic tapered sheath. After a 0.025-inch guidewire had been placed, the tapered sheath was inserted through the stenosis site to a point near the fractured stent. After the inner catheter had been removed, a 1.8-mm diameter biopsy forceps (Radial Jaw 4 pediatric biopsy forceps; Boston Scientific Japan, Tokyo, Japan) was inserted through the lumen of the indwelling outer sheath and the fractured stent was grasped ( Video 1 ). Although the stent could not be pulled into the outer sheath because of its distal flap, it was successfully retrieved by pulling it out along with the sheath ( Fig. 4 ).

**Fig. 2:**
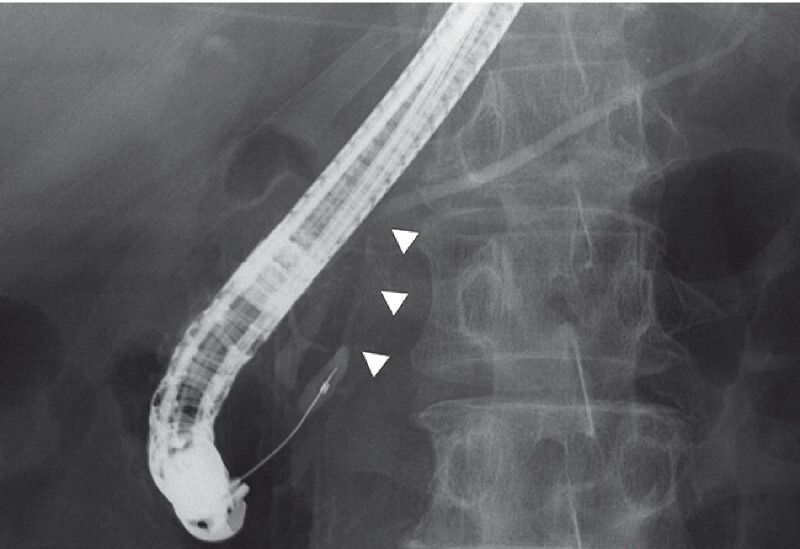
Fluoroscopic view during endoscopic retrograde pancreatography showing severe stenosis of the main pancreatic duct from the pancreatic head to the body (white arrowhead) that was caused by chronic pancreatitis.

**Fig. 3:**
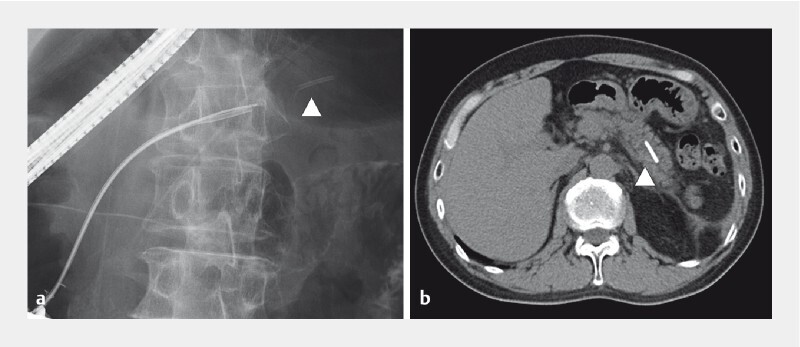
The fractured migrated stent (white arrowhead) is visible in the proximal pancreatic duct on: **a** fluoroscopic pancreatography; **b** computed tomography.

**Video 1** Successful retrieval of a fractured migrated pancreatic stent using an endoscopic tapered sheath.


**Fig. 4:**
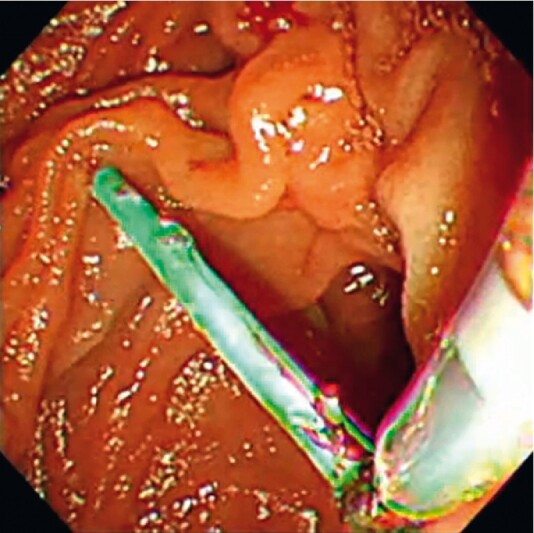
Endoscopic image showing the successfully retrieved fractured pancreatic stent that had been pulled out into the duodenum along with the outer sheath.

To the best of our knowledge, this is the first report of the use of a tapered sheath to retrieve a migrated fractured pancreatic stent. This method could improve the outcome of fractured migrated stent removal, even in cases where severe pancreatic duct stenosis is present.

Endoscopy_UCTN_Code_CPL_1AK_2AD

## Notes

Endoscopy E-Videos

https://eref.thieme.de/e-videos
*E-Videos* is an open access online section of the journal *Endoscopy* , reporting on interesting cases and new techniques in gastroenterological endoscopy. All papers include a high-quality video and are published with a Creative Commons CC-BY license. Endoscopy E-Videos qualify for HINARI discounts and waivers and eligibility is automatically checked during the submission process. We grant 100% waivers to articles whose corresponding authors are based in Group A countries and 50% waivers to those who are based in Group B countries as classified by Research4Life (see: https://www.research4life.org/access/eligibility/ ). This section has its own submission website at https://mc.manuscriptcentral.com/e-videos
